# A Longitudinal, Observational Analysis of Neuronal Injury Biomarkers in a Case Report of a Patient With Paraneoplastic Anti-CRMP5 Antibody-Associated Transverse Myelitis

**DOI:** 10.3389/fneur.2021.691509

**Published:** 2021-07-16

**Authors:** Christopher Mizenko, Jeffrey L. Bennett, Gregory Owens, Timothy L. Vollmer, Amanda L. Piquet

**Affiliations:** ^1^Department of Neurology, University of Colorado, Aurora, CO, United States; ^2^Department of Ophthalmology, University of Colorado Anschutz Medical Campus, Aurora, CO, United States

**Keywords:** paraneoplastic, CRMP5 antibody, biomarker, neurofilament light, myelitis

## Abstract

Biomarkers are needed to guide therapeutic decision making in autoimmune and paraneoplastic neurologic disorders. Here, we describe a case of paraneoplastic collapsing response-mediator protein-5 (CRMP5)-associated transverse myelitis (TM) where plasma neurofilament light (NfL) chain and glial fibrillary protein (GFAP) levels were observed over a 14-month clinical course, correlating with radiographical and clinical outcome measures in response to treatment. Blood and CSF samples obtained at diagnosis as well as 7 and 14 months into treatment. At the time of initial diagnosis, both plasma NfL (782.62 pg/ml) and GFAP (283.26 pg/ml) were significantly elevated. Initial treatment was with IV steroids and plasma exchange (PLEX) followed by neuroendocrine tumor removal, chemotherapy, and radiation. After initial improvement with chemotherapy, the patient experienced clinical worsening and transient elevation of plasma NfL (103.27 pg/ml and GFAP (211.58 pg/ml) levels. Whole body positron emission tomography PET scan did not demonstrate recurrence of malignancy. Repeat PLEX and rituximab induction resulted in improvements in patient function, neurologic exam, and plasma biomarker levels. To our knowledge, this is the first described longitudinal, prospective analysis of neuronal injury biomarkers and association of clinical treatment outcomes in CRMP5 myelitis. Our findings suggest that clinical improvement correlates with NfL and GFAP concentrations.

## Introduction

### Neuronal Injury Biomarkers

Identification and quantification of neuroaxonal damage may improve diagnostic accuracy and treatment of autoimmune and paraneoplastic neurological disease. Biomarkers currently under investigation for monitoring neuronal injury, demonstrate promise for use in multiple clinical settings (1). The cytoskeleton of central nervous system (CNS) neurons is composed of intermediate neurofilaments (Nfs) composed of four heterologous polypeptidens: alpha-internexin, neurofilament light chain (NfL), neurofilament medium chain (NfM), and neurofilament heavy chain (NfH). Following neuronal injury, the concentrations of these neurofilament proteins are elevated in cerebral spinal fluid (CSF) and blood. Most, if not all neurodegenerative diseases will result in an elevation in blood Nf levels, providing a general indicator of axonal damage. Since NfL is more highly expressed than other Nf polypeptides, Nfl has now become the preferential candidate biomarker for following axonal injury (1).

Other neuronal biomarkers may provide alternative information on CNS injury in neuro-immunological disorders. A highly localized cytosolic protein in neurons and neuroendocrine cells, ubiquitin carboxy-terminal hydrolase L1 (UCH-L1), is involved in neuronal repair after injury through removal of abnormal proteins *via* the ubiquitin-proteasome pathway, autophagy, and regulation of synaptic function. Indeed, prior research has demonstrated a correlation between levels of UCH-L1 and intracranial injury (2).

Glial fibrillary acidic protein (GFAP) is a brain-specific intermediate filament expressed in astrocytes and ependymal cells. Increased levels of GFAP may reflect astrogliosis, upregulated expression, glial scarring, and astrocyte destruction (3–5). Serum levels of GFAP and NL are likely to be good biomarkers for disease activity and disability in neuromyelitis optic spectrum disorders (NMOSD) (6). Furthermore, in patients enrolled in N-MOmentum trial (NCT2200770), serum GFAP levels increased significantly within 1 week of an NMOSD attack in placebo-treated patients (5). In multiple sclerosis (MS), studies propose GFAP as a biomarker of disease progression (7, 8) based on observation as a correlate between GFAP levels in CSF and neurologic disability assessed by Expanded Disability Status Score (EDSS) and Multiple Sclerosis Severity Scale (MSSS) (9). Additional reports of serum GFAP levels as brain-specific marker for malignant gliomas (10).

Tau protein is a heat stable, microtubule-associated protein (MAP) essential for inducing microtubules from tubulin and stabilization of cytoskeleton scaffolding; the absence of tau *in vitro* prevents tubulin from assembling into microtubules (11, 12). One main function of tau is the stabilization and flexibility of axonal microtubules; abnormal tau deposition is common in neurodegenerative diseases (12). In Alzheimer's disease (AD) and dementia, both tau and beta-amyloid pathology, have been correlated with blood NfL levels (13). A large prospective study showed that plasma tau levels associate with worsening cognition, atrophy, and hypometabolism during follow-ups despite no clear association between tau and NfL (13). However, increases of plasma NfL concentrations in combination with reduced plasma beta-amyloid strongly associated with higher risk of developing dementia and AD dementia (13).

### Paraneoplastic Neurological Disorders

Paraneoplastic neurological disorders (PNDs) are immune-mediated disorders that can affect any part of the neuroaxis and occur in association with cancer. This is thought to be driven by the immune response directed against proteins shared between tumor cells and neurons, thus resulting in neuronal destruction and cell death. PNDs are characterized by the detection of neuronal autoantibodies in the serum and CSF. One such autoantibody is anti-CV2/collapsing response mediator protein (CRMP)5. This protein is integral to the morphology of neurons and is highly expressed in cerebellar purkinje cells. CRMP5 neuronal antibody is often associated with lung cancer and thymoma-related autoimmunity (14). Clinical manifestations may vary and include rapidly progressive myelopathy, cerebellar ataxia, polyneuropathy, optic neuritis, and chorea (15–18). The management of CRMP5 autoimmunity focuses on diagnosing and treating the underlying tumor in combination with immunotherapy.

Since PND-like CRMP5 often results in progressive and debilitating symptoms, early recognition is crucial to minimize irreversible neurological damage. While clinical assessments are the sole measure for assessing therapeutic response; however, novel quantitative biomarkers are needed to monitor the immune response and neuronal injury. Such biomarkers will be critical for both patient care and the design of robust randomized controlled trials.

In this longitudinal evaluation of a single female patient with anti-CRMP5-associated transverse myelitis (TM), we assessed four plasma markers of neuronal injury including NfL, GFAP, tau, and UCH-L1 in relation to neurologic symptoms and radiographic disease activity during her clinical and treatment course.

## Methods

The patient was enrolled in the Autoimmune, Paraneoplastic, and Inflammatory Neurological Disease (APIND) Patient Registry at the University of Colorado. The study was approved by the Institution Review Board of the University of Colorado, Aurora, CO. As part of the APIND, clinical data and biorepository specimens were collected prospectively over time at each follow-up visit. Biorepository specimens collected included CSF at the time of diagnosis as well as longitudinal serum samples. Samples are stored at −80°C.

Patient specimens were collected at the time of clinical presentation (time point 0), as well as 6 and 14 months into her clinical course. Sample collection at time point 0 occurred prior to the administration of any therapeutic agents including the initiation of plasma exchange.

Serum and CSF levels of tau, GFAP, UCH-L1, and Nfl were measured using the Quanterix Small Immunoassay (Simoa) Neurology 4-PlexA assay. Normal values were provided by Quanterix for plasma, serum, and CSF (Supplementary Table 1) (19). The assay performed for internal control and quality assurance of Quanterix machine and N4PA. Using Person sample correlation coefficient, self-validation yielded high correlation between the two plasma samples of 0.998651. Adjusting for the small sample size *R*-squared was 0.997303.

## Results

### Clinical Case Report

**Figure 1:**
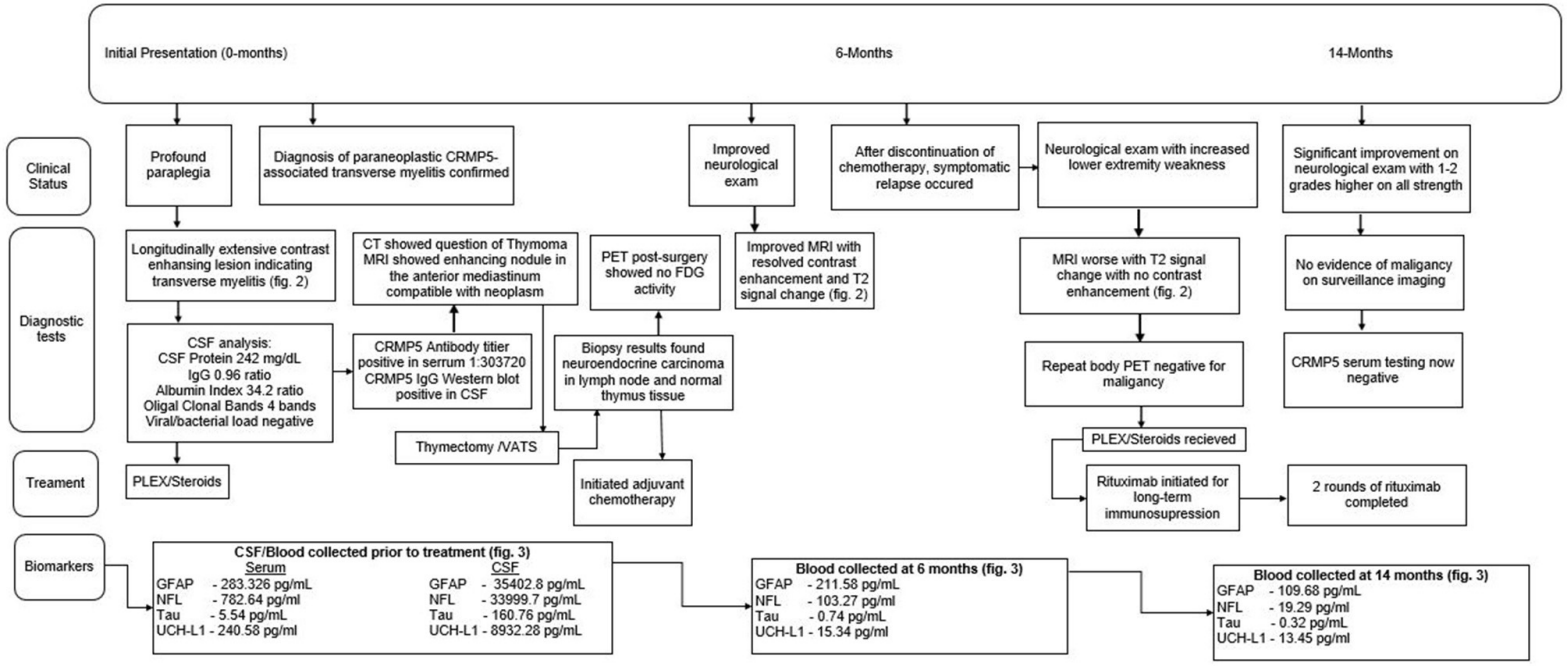
Timeline of clinical course, diagnostic testing, treatment, and biomarker sampling.

A 54-year-old female with a 39-pack-year history of tobacco use and a history of rheumatoid arthritis presented to an outside hospital with a history of progressive lower extremity weakness (Figure 1 for timeline of events). Her recent history was notable for worsening back pain over the prior 5 months. She denied any sensory loss but endorsed urinary frequency and hesitation. She had an abrupt worsening of her motor weakness occurring over days leading to transfer to our institution.

Initial neurological exam was remarkable for profound lower extremity weakness (0/5) throughout, with the exception of 1/5 dorsiflexion in the right ankle and bilateral toes. In the upper extremities, she had full strength on the right and mild extensor weakness on the left (4/5). She had diffuse hyperreflexia, more profound and on the right, with bilateral clonus and Hoffmann's sign.

**Figure 2:**
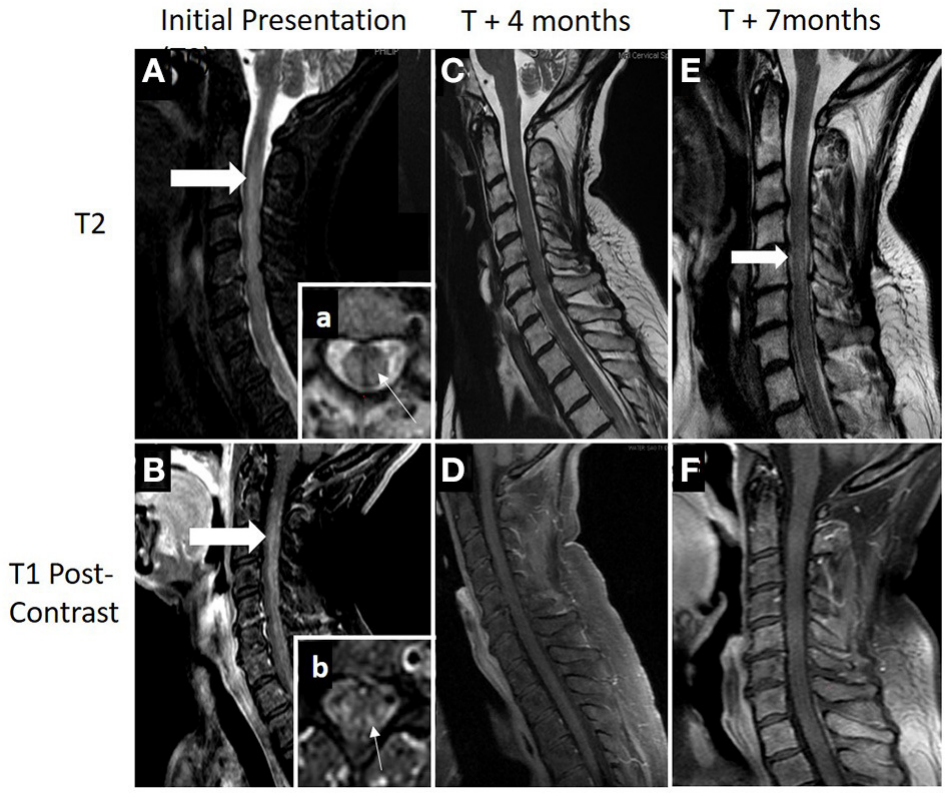
**(A,B)** MRI demonstrating an longitudinally extensive T2 lesion (top row; thick white arrow) with associated contrast enhancement (bottom row; thick white arrow). Box (a), axial view showing a typical presence of transverse myelitis involving a central lesion, involving more than 2/3 of the cross-sectional area (thin white arrow). Box (b), areas of patchy contrast enhancement concentrated within areas of the posterior cord (thin white arrow). **(C,D)** Patient had clinical and radiographical improvement at 4 months after diagnosis following acute treatment with plasma exchange, high-dose steroids, and adjuvant chemotherapy. **(E,F)** Seven months after diagnosis and 1 month following completion of her adjuvant chemotherapy, she was hospitalized again for worsening of lower extremity weakness and pain. Repeat cervical spine MRI showed some worsening T2 signal change within the cord (thick white arrow) with no evidence of contrast enhancement. Acute therapy with plasma exchange and intravenous steroids were given over 5 days followed by rituximab induction (1,000 mg on days 0 and 14). Additional cancer screening with full body PET scan remained negative with clinical relapse.

Magnetic resonance imaging (MRI) showed a contrast-enhancing, longitudinally extensive lesion from C3 down to T12 (Figure 2). Brain MRI was unremarkable.

CSF analysis demonstrated a lymphocytic predominant pleocytosis of 93 nucleated cells/μl [reference range (ref): <5 cells/μl], elevated protein of 242 mg/dl (ref: <45 mg/dl), elevated immunoglobulin-G (IgG) index of 0.96 (normal ratio: 0.28–0.66), and four CSF-restricted oligoclonal bands (OCBs). Infectious work up on the CSF was negative for herpes simplex virus (HSV), varicella zoster virus (VSV), arbovirus panel (including West Nile virus), and enterovirus.

The patient's history, neurological exam, and radiographic findings consistent with transverse myelitis, with a high degree of suspicion for autoimmune or paraneoplastic etiology including neuromyelitis optic spectrum disorder (NMOSD) or other inflammatory etiology such as neurosarcoidosis. Infectious etiologies have largely been ruled out though CSF-specific PCR and antibody testing as above. While awaiting antibody results, she was empirically treated with a 5-day course of intravenous (IV) methylprednisolone (MP) and plasma exchange (PLEX).

Her aquaporin-4 (AQP4) eventually returned negative as well as myelin oligodendrocyte glycoprotein (MOG). CRMP5 antibody returned positive in serum 1:30,720 (ref: <1:240 titer) and CRMP5 IgG Western Blot CSF Positive (Mayo Clinic Laboratories), thus, leading to a final diagnosis of paraneoplastic CRMP5-associated transverse myelitis.

Further malignancy work up with computed tomography (CT) of chest showed two inferior left thyroid lobe nodules, largest 8 mm and normal right thyroid. Soft tissue density nodule in anterior mediastinum adjacent to left brachycephalic vein and lungs indicated mild centrilobular emphysema. Biopsy results of the mediastinum nodule found the tumor to be high-grade neuroendocrine carcinoma, entirely contained in single lymph node without extranodal extensions or involvement of thymic tissue (Ki67: >90%). Follow-up positron emission tomography (PET)-CT postsurgery showed no occult flourodeoxyglucose (FDG) avidity.

Despite the inability to identify a primary tumor, given the strong association of CRMP5-IgG with neuroendocrine tumors, specifically small cell lung cancer, she started on adjuvant chemotherapy with etoposide. Two months after treatment initiation, patient's neurologic exam showed improvements with lower extremity weakness noted having antigravity movements in knee flexion and extension.

Outpatient follow-up visit occurred 5 months after presentation. She was now able to stand and transfer but still not able to walk with assistance. Motor exam continued to demonstrate improvement with the ability to move all muscle groups against gravity with proximal greater than distal weakness remaining. Repeat spinal imaging demonstrated resolution of contrast enhancement and improved T2 signal change (Figure 2).

Seven months after her initiation presentation she was readmitted to the hospital for worsening pain and weakness in the lower extremities. Neurologic exam demonstrated increased weakness, primarily in the left leg and arm.

MRI showed longitudinally extensive T2 signal abnormality within medial cord from C2 through T1 with no accompanying gadolinium enhancement. Repeat PET-CT showed no evidence of FDG-avid malignancy. Following PLEX and IVMP over 5 days, there was improvement in her strength. Given her clinical relapse, rituximab was initiated with induction dose at 1,000 mg on days 0 and 14 and the patient initiated a 4-week course of radiation therapy. She has since completed two cycles of rituximab infusions. Her oncologist continues to monitor for cancer, and her surveillance screen remains negative.

Fourteen months after initial visit, neurological examination significantly improved with one to two grades higher on all strength testing and she was able to ambulate with assistance using her walker, although she still must rely on wheelchair the majority of the time. Repeat CRMP5 antibody titer was now negative.

From the patient's perspective, she has had significant benefit after PLEX and chemotherapy but felt the discontinuation of chemotherapy had brought back some of her ongoing neuropathic pain symptoms and weakness. With the initiation of rituximab, most of the symptoms have subsided; however, mobility remains a challenge, but this is much better than her initial presentation. Her only ongoing bothersome symptom is lower back pain, potentially multifactorial involving spasms, degenerative disks, and neuropathic pain.

### Neuronal Marker Results

See Supplementary Material for summary of neuronal marker results for each time point including Supplementary Table 2 (0 months), Supplementary Table 3 (6 months), and Supplementary Table 4 (14 months).

#### 0 Months (All Samples Collected Prior to Intervention With Empiric Immune Therapies)

Blood and spinal fluid collected at initial presentation showed significant elevation of all aforementioned biomarkers (see Supplementary Table 1 for normal control values). Most notable increase was in the CSF. The universal axonal injury biomarker NfL CSF level 3,399.7 (control median: 1,241 pg/ml) and plasma was 782.62 pg/ml. Concomitantly elevated CSF levels of GFAP were observed in the CSF 35,402.8 (control median: 14,624 pg/ml) and plasma elevated at 283.32 pg/ml. CSF levels of UCH-L1 elevated at 8,932.28 pg/ml (control median: 989 pg/ml) and plasma was 240.58 pg/ml (no normal control data). Additionally, tau CSF elevated to 160.77 pg/ml (control median: 118 pg/ml) and plasma at 5.54 pg/ml (control median: 2.21 pg/ml).

#### 6 Months

NfL plasma levels remained elevated but significantly reduced at 103.27 pg/mL. GFAP was 211.58 pg/mL, UCH-L1 15.34, and tau normalized at 0.73 pg/ml.

#### 14 Months

**Figure 3:**
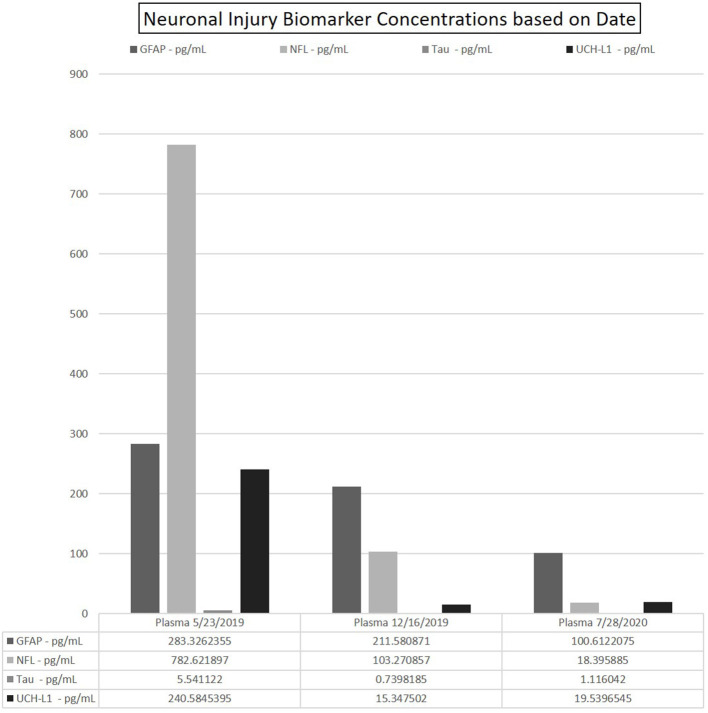
Data depicting the fitted concentration values for GFAP, NFL, Tau, and UCH-L1 at time points 0–6–14 months. Date of sample collection utilized to represent these time points. Each run yielded 2-well readings on the N4PA; the averaged of each shown for clarity. CSF omitted from table due to exceedingly high concentrations of NfL as compared with the values in the patients' blood.

Plasma NfL levels reduced to near normalization of 18.40 pg/mL. GFAP was 100.61 pg/mL, UCH-L1 19.53, and tau normalized at 1.12 pg/ml. See trend in Figure 3.

## Discussion

To our knowledge, this is the first case report of CRMP5-associated TM with serial collection of NfL and other CNS-derived proteins in which concentrations tended to correlate with clinical symptoms. Here, we provided an observation of four novel quantitative biomarkers in this patient's 14-month clinical course.

We noted an elevation of 2,640% of CSF concentrations of NfL over normative control values, potentially reflecting disease activity. Prior studies of inflammatory demyelinating diseases noted increased CSF NfL concentrations across various groups including clinically isolated syndrome (CIS; mean NfL: 15,138.50 pg/ml), NMOSD (mean: 15,400.00 pg/ml), and MS (mean: 5,789.50 pg/mL) (20). This study suggests no statistically significant association with classical biomarkers (OCB+/– in CIS and MS or AQP4 in NMOSD) and NfL concentrations but a potential relationship between CSF NfL level and disease activity represented by MRI contrast-enhancing lesions (20). Analogous associations in axonal injury biomarkers and imaging for traumatic brain injury (TBI) patients has also indicated potential clinical significance. Plasma GFAP concentrations may complement MRI as a biomarker for intracranial lesions undetectable on CT with the possible adjunct providing differentiation to traumatic pathology based on relative differences in GFAP concentration (21). Similar trends appear in our case of CRMP5-associated TM and MRI lesion enhancement accompanied with elevated neuronal biomarkers suggestive of the potential correlation. Blood-brain barrier (BBB) leakage, also delineated by enhancement on MRI, may be important for the detection of these biomarkers in the plasma.

In addition to NfL, our patient had an initial CSF GFAP concentration of 142% above normative control values. GFAP is considered to reflect the sum of degraded astrocytes in CSF and associations with NfL levels may denote clinical outcomes and/or relapse in MS (9, 22) and NMOSD (6). Additionally, there was an 803% increase in UCH-L1 and a mild elevation of tau. Initial plasma concentrations were elevated for all four biomarkers; this increase in blood concentrations were congruent with her elevations in CSF.

Markers of neuronal damage may potentially aid clinicians in predictions of patient outcome, nonrecovery deficits, and treatment efficacies. Studies in relapsing remitting MS (RRMS) have shown that NfL is most elevated during an attack, and as duration from attack increases, NfL decrease (23). In this case, there was drastic improvement in her neurologic exam and MRI after PLEX and steroids alone. This rapid improvement suggests early and aggressive intervention may ensure the best possible clinic outcome. Furthermore, this may suggest that early treatment could offer the least amount of neuronal damage from CRMP-5-associated TM as her markers of neuronal damage were significantly elevated at the time of her diagnosis and subsequently decreased over time. After discontinuation of chemotherapy, symptomatic relapse occurred 6 months after initial presentation. Neurologic exam indicated improvements compared with initial presentation; however, during physical therapy, there was notable decreased stamina and fatigue. Repeat MRI showed no enhancement (Figure 2D) and PET/CT was negative for malignancy. Additionally, as shown in Figure 3, plasma NfL, GFAP, and UCH-L1 levels were greatly reduced, yet remained elevated compared with normative values. Plasma UCH-L1 dramatically reduced from 240.58 to 15.34 pg/ml and tau normalized. Interestingly, plasma NfL dramatically decreased while there was only a modest reduction in GFAP. Currently, GFAP evaluation as a marker in gauging TBI has indicated higher initial GFAP with a biphasic release in serum with level decreasing during the first 6 months but increasing over subsequent visits postinjury, which may correspond to our patient and her maintaining elevated levels (24). Previous MS studies evaluating GFAP as a biomarker in disease progression indicated augmented GFAP levels in MS patients, noting strong correlation between GFAP in CSF and neurological disability during MS progression (9). After adjusting for age, GFAP and NfL increased compared with healthy controls but GFAP was only statistically significant from examinations 8–10 years apart indicating the potential use GFAP in the neurodegenerative process (9). Therefore, continuous monitoring of NfL and GFAP in tandem may provide further indications of not only initially sustained damage and treatment effectiveness but disease progression in CRMP5-associated TM patients.

Additionally, as the 6-month sample collection was conducted during a clinical relapse, this NfL reduction of 86.80% stipulates that neuronal damage severity may have been hindered with acute immunotherapies and treatment of her underlying malignancy. Moreover, given that her relapse occurred upon discontinuation of chemotherapy, a potential for degree of biomarker elevation might provide a prognostic indicator toward the necessity of long-term immunosuppression.

Following treatment with rituximab, at 14 months past initial time point, the patient showed significant clinical improvements and improved strength. Plasma NfL and GFAP at this point were only mildly elevated and the reduced plasma concentration correlated with neurologic exam and radiographic improvements. This observed correlation suggests monitoring these biomarkers may be useful to quantify disease severity, progression, and treatment efficacy. Notably, plasma UCH-L1 remained elevated with a slight increase to 19.38 pg/ml (from 15.35 at 6 months), albeit dramatically decreased from initial presentation (240.58 pg/ml).

Our observed drastic elevation of neuronal injury biomarkers at disease onset might further indicate damage intensity due to mechanisms of action and inflammatory response in this case. The autoimmune response to intracellular antigens, like CRMP5, may cause damage indirectly by immune complex formation, immune activation, and other processes (25). However, the role of antibodies causing neuronal injury remains controversial. Antibodies against intracellular targets are not thought to be directly pathogenic, and rather, likely mediate damage *via* cytotoxic T cells; although, there has been some evidence to dispute this assumption (26, 27). PNDs associate with intracellular antibodies are often characterized by irreversible, neuronal death and can be refractory to immune therapy. Larger, longitudinal prospective studies of neuronal injury biomarkers in CRMP5 autoimmunity and other PNDs may provide some insight in our understanding of disease pathogenesis and approaches to treatment.

## Conclusion

This longitudinal analysis of neuronal injury biomarkers in CRMP5-associated TM provides a case example of the possible utility of these biomarker with regard to disease activity and treatment response in PNDs. We observed neurologic improvements and the absence of contrast enhancement on subsequent MRIs that correlated with the reduction of NfL and GFAP concentrations. Similar trends have been reported in other neurological diseases. Further understanding is required regarding the relationship of these neuronal markers and their association with neurologic outcomes and treatment response, yet the speculative mechanisms proposed may provide insight for future research. A strong need for randomized, controlled clinical trials to evaluate the efficacy of immunotherapy in autoimmune and paraneoplastic neurological disease exists. Using neuronal injury biomarkers may provide a valuable outcome-surrogate marker and should be explored to assist in the development of future clinical trials.

## Data Availability Statement

The original contributions presented in the study are included in the article/Supplementary Material, further inquiries can be directed to the corresponding author/s.

## Ethics Statement

Written informed consent was obtained from the individual for the publication of any potentially identifiable images or data included in this article.

## Author Contributions

All authors listed have made a substantial, direct and intellectual contribution to the work, and approved it for publication.

## Conflict of Interest

JB reports personal fees from Roche, personal fees from Genentech, personal fees from Viela Bio, personal fees from Chugai Pharma, personal fees from Alexion, grants and personal fees from Novartis, personal fees from Genzyme, personal fees from Teva Neuroscience, grants and personal fees from EMD Serono, personal fees from Frequency Therapeutics, personal fees from Equillium, personal fees from Clene Nanoscience, personal fees from Mitsubishi-Tanabe, personal fees from Reistone Bio, grants from National Institutes of Health, grants from Guthy Jackson Charitable Foundation, and grants from National Multiple Sclerosis Foundation, outside the submitted work. In addition, JB has a patent for aquaporumab issued. TV has received compensation for lectures and consultancy from Biogen, Genentech/Roche, Siranax, Celgene, EMD Serono, and Novartis and has received research support from Rocky Mountain Multiple Sclerosis Center, Celgene, Biogen, Anokion, Genentech, F. Hoffmann-La Roche Ltd, GW Pharma, and TG Therapeutics, Inc. AP has received research funding from the University of Colorado and Rocky Mountain MS Center, consulting fees from Genentech/Roche and Alexion, and honorarium from MedLink and publication royalties from Springer. The remaining authors declare that the research was conducted in the absence of any commercial or financial relationships that could be construed as a potential conflict of interest.
