# Supplementary material for: A Longitudinal, Observational Analysis of Neuronal Injury Biomarkers in a Case Report of a Patient With Paraneoplastic Anti-CRMP5 Antibody-Associated Transverse Myelitis
Source: Front Neurol. 2021 Jul 16;12:691509. doi: 10.3389/fneur.2021.691509 (PMC8328144; doi:10.3389/fneur.2021.691509)
Supplement: Supplementary file 1 [file Data_Sheet_1.docx]

**Supplementary Material**

**Table 1 Normal Values for Neurology 4-PlexA Assay**

| Target | Sample Type | Median Conc. (pg/mL) |
| --- | --- | --- |
| NfL | EDTA Plasma | 10.6 |
|  | Serum | 10.8 |
|  | CSF | 1241 |
| GFAP | EDTA Plasma | 89.7 |
|  | Serum | 90.2 |
|  | CSF | 14624 |
| UCH-L1 | EDTA Plasma | * |
|  | Serum | * |
|  | CSF | 989 |
| Tau | EDTA Plasma | 2.21 |
|  | Serum | 0.861 |
|  | CSF | 118 |

Neurology 4-PlexA assay (N4PA) kits utilized to obtain target biomarkers of total tau, GFAP, UCH-L1, and NfL. Normal values provided by Quanterix endogenous sample readings for plasma, serum, and CSF (n=20 each for all markers except UCH-L1 with n=10 plasma and serum samples and n=5 CSF samples). * indicates no values above lower limit of quantification (LLOQ), the lowest amount of analyte that can be quantified with accuracy(19).

**Table 2:**

| Time | Bead Plate | Type | Fitted Conc. (pg/mL) |
| --- | --- | --- | --- |
| 0-months | NFL | Plasma | 782.62 |
| 0-months | GFAP | Plasma | 283.32 |
| 0-months | UCH-L1 | Plasma | 240.58 |
| 0-months | Tau | Plasma | 5.54 |
| 0-months | NFL | CSF | 33999.70 |
| 0-months | GFAP | CSF | 35402.80 |
| 0-months | UCH-L1 | CSF | 8932.28 |
| 0-months | Tau | CSF | 160.77 |

**Table 3:**

| Time | Bead Plate | Type | Fitted Conc. (pg/mL) |
| --- | --- | --- | --- |
| 6-months | NFL | Plasma | 103.27 |
| 6-months | GFAP | Plasma | 211.58 |
| 6-months | UCH-L1 | Plasma | 15.35 |
| 6-months | Tau | Plasma | 0.739 |

**Table 4:**

| Time | Bead Plate | Type | Fitted Conc. (pg/mL) |
| --- | --- | --- | --- |
| 14-months | NFL | Plasma | 18.40 |
| 14-months | GFAP | Plasma | 100.61 |
| 14-months | UCH-L1 | Plasma | 19.54 |
| 14-months | Tau | Plasma | 1.12 |
